# Comparative Efficacy and Tolerability of Tirzepatide Versus Semaglutide at Varying Doses for Weight Loss in Non-diabetic Adults With Obesity: A Network Meta-Analysis of Randomized Controlled Trials

**DOI:** 10.7759/cureus.90335

**Published:** 2025-08-17

**Authors:** Alousious Kasagga, Delvy Rebellow, Tooba Hashmi, Malik Y Husami, Pooja Lama, Kaavya Arul Selvan, Kevin Nakasagga

**Affiliations:** 1 Pathology, Peking University, Beijing, CHN; 2 Internal Medicine, Malankara Orthodox Syrian Church Medical College, Kolenchery, IND; 3 Internal Medicine, California Institute of Behavioral Neurosciences and Psychology, California, USA; 4 Internal Medicine, Dow Medical College, Karachi, PAK; 5 Orthopedics, University Hospitals of Leicester NHS Trust, Leicester, GBR; 6 Anesthesiology and Critical Care, Nepalese Army Institute of Health Sciences, Kathmandu, NPL; 7 Anesthesiology and Critical Care, Government Medical College, Omandurar, Chennai, IND; 8 Anesthesiology, Milwaukee School of Engineering, Milwaukee, USA

**Keywords:** dose response, gip/glp-1 agonist, meta-analysis, network meta-analysis, non-diabetic adults, obesity treatment, semaglutide, systematic review and meta-analysis, tirzepatide, weight loss therapy

## Abstract

Obesity in non-diabetic adults remains a significant clinical challenge, prompting the need for effective pharmacological therapies that achieve meaningful weight loss with acceptable tolerability. Semaglutide, a selective glucagon-like peptide-1 (GLP-1) receptor agonist, and tirzepatide, a dual GLP-1 and glucose-dependent insulinotropic polypeptide (GIP) receptor agonist, have shown promise in recent trials. However, direct comparisons across their dose ranges are lacking.

This systematic network meta-analysis (NMA) compared multiple doses of tirzepatide (5 mg, 10 mg, 15 mg, and maximum tolerated dose (MTD)) and semaglutide (2.4 mg and MTD) versus placebo in non-diabetic adults with obesity. Eight randomized controlled trials comprising 7,179 participants were included. The primary outcome was the percentage change in body weight. Secondary outcomes included changes in waist circumference, the proportion achieving ≥15% weight loss, and safety outcomes such as discontinuation due to adverse events and gastrointestinal side effects. A random-effects model was used for all analyses, and small-study effects were assessed using comparison-adjusted funnel plots and Egger-type regression.

All active interventions resulted in significantly greater weight loss compared to placebo. Tirzepatide MTD demonstrated the greatest effect (mean difference (MD): −20.90%; 95% confidence interval (CI): −24.93 to −16.87), followed by tirzepatide 15 mg (MD: −18.08%; 95% CI: −20.38 to −15.78), tirzepatide 10 mg (MD: −14.93%; 95% CI: −17.23 to −12.63), semaglutide MTD (MD: −14.40%; 95% CI: −19.82 to −8.98), semaglutide 2.4 mg (MD: −11.78%; 95% CI: −13.91 to −9.64), and tirzepatide 5 mg (MD: −11.49%; 95% CI: −14.65 to −8.34). Similar dose-dependent effects were observed for waist circumference and the likelihood of achieving ≥15% weight loss. The certainty of evidence for this ≥15% weight loss outcome, assessed using the Grading of Recommendations Assessment, Development, and Evaluation (GRADE) framework applied to our NMA estimate threshold, was high for most interventions, except moderate for semaglutide MTD and tirzepatide 5 mg. The highest odds of treatment discontinuation due to adverse events were observed with semaglutide MTD (odds ratio (OR): 7.36; 95% CI: 2.56 to 21.15) and tirzepatide MTD (OR: 5.56; 95% CI: 2.28 to 13.58). Gastrointestinal side effects were more common with higher-dose regimens. No publication bias was detected.

Tirzepatide at 15 mg and MTD offers the greatest efficacy for weight loss in non-diabetic adults with obesity, though at the cost of increased gastrointestinal side effects and higher discontinuation rates. These findings support a clear dose-response relationship and underscore the importance of tailoring treatment decisions to individual patient tolerability and goals.

## Introduction and background

Obesity in non-diabetic adults is a major public health issue linked to higher risks of cardiovascular disease, metabolic complications, and reduced quality of life [1,2]. While lifestyle changes are the cornerstone of treatment, many individuals struggle to achieve and sustain meaningful weight loss, which highlights the need for effective medical therapies [3].

Glucagon-like peptide-1 (GLP-1) receptor agonists, especially semaglutide, and dual GLP-1/glucose-dependent insulinotropic polypeptide (GIP) receptor agonists, such as tirzepatide, have emerged as key pharmacological options in recent years [4]. Semaglutide, approved for long-term weight management, has shown strong efficacy in the Semaglutide Treatment Effect in People with obesity (STEP) trials, consistently leading to significant weight reduction. Tirzepatide, originally developed for the treatment of type 2 diabetes, has demonstrated promising results for weight loss in the SURpass tirzepatide Obesity Multi-dose evalUaTion (SURMOUNT) trials, particularly at the 15 mg and maximum tolerated dose (MTD) regimens [5].

Until recently, there were no direct comparisons between semaglutide and tirzepatide, leaving important questions about their relative efficacy, dose-response relationships, and safety profiles unanswered [6]. The SURMOUNT-5 trial offered the first head-to-head evaluation of the two drugs at their respective MTDs. However, this trial alone does not fully capture the range of tirzepatide dosing strategies explored in the broader literature. Furthermore, differences in tolerability, especially gastrointestinal side effects and treatment discontinuation, are still not clearly understood.

To fill these knowledge gaps, we conducted a comprehensive network meta-analysis (NMS) comparing the effectiveness and tolerability of different doses of tirzepatide and semaglutide in non-diabetic adults with obesity. Our primary outcomes included percent weight reduction, the proportion achieving at least 15% weight loss, rates of treatment discontinuation, and gastrointestinal adverse events. The results aim to inform clinical practice and guideline development, offering insights into optimal pharmacologic choices for obesity management.

## Review

Methods

This systematic review and network meta-analysis followed the Preferred Reporting Items for Systematic Reviews and Meta-Analyses extension for Network Meta-Analyses (PRISMA-NMA) guidelines [7].

Eligibility Criteria

We included randomized controlled trials (RCTs) that enrolled non-diabetic adults (18 years or older) with overweight or obesity, defined as a body mass index (BMI) of 27 kg/m² or higher. Eligible studies evaluated subcutaneous tirzepatide at 5 mg, 10 mg, 15 mg, or a participant-specific MTD, defined as dose escalation to either 10 mg or 15 mg based on tolerability, or semaglutide at 2.4 mg or its own MTD (escalated to 1.7 mg or 2.4 mg based on tolerability). Trials were eligible if they compared any of these interventions with a placebo or with each other and reported at least one of the prespecified outcomes. We included studies regardless of location or blinding status.

We excluded trials that included participants with type 1 or type 2 diabetes, as well as studies of oral semaglutide, due to differences in drug formulation and target populations. Non-randomized studies, observational cohorts, and single-arm trials were excluded from the analysis. When multiple reports of the same trial were available, we used the most complete or primary publication for data extraction.

Search Strategy and Study Selection

We conducted a systematic search of PubMed, the Cochrane Central Register of Controlled Trials (CENTRAL), and ClinicalTrials.gov to identify eligible RCTs published between January 1, 2000, and June 1, 2025. The search strategy combined Medical Subject Headings (MeSH) and free-text terms related to the population ("obesity" OR "overweight"), interventions ("tirzepatide" OR "semaglutide"), and study design ("randomized controlled trial" OR "placebo-controlled"), using Boolean operators. Example syntax included the following: ("obesity"[MeSH Terms] OR "overweight"[MeSH Terms]) AND ("tirzepatide" OR "semaglutide") AND ("randomized controlled trial"[Publication Type] OR "placebo"). We applied filters to include only English-language studies published in peer-reviewed journals to ensure methodological quality and consistency in outcome reporting.

Two reviewers independently screened all titles and abstracts, followed by full-text reviews based on predefined inclusion criteria. Disagreements were resolved through discussion and consensus. We also manually reviewed reference lists of included articles and relevant systematic reviews to capture any additional eligible studies. For trials with multiple publications, we used the most complete or longest follow-up report available.

Data Extraction and Risk of Bias

Two reviewers independently extracted data utilizing a structured spreadsheet developed in Microsoft Excel (Microsoft Corporation, Redmond, Washington, United States; 2018), following Cochrane guidelines. We collected details on study characteristics (first author, publication year, trial name, sample size, study duration, and geographic region), population characteristics (mean age, sex distribution, and baseline BMI), intervention details (drug name, dose, dosing schedule, and duration), comparator treatments, and all prespecified outcomes. When numerical values were not directly reported, we extracted them from figures or calculated them using standard methods recommended in the Cochrane Handbook for Systematic Reviews of Interventions [8]. Different opinions among the reviewers were addressed through discussion until an agreement was achieved.

Risk of bias was assessed independently by both reviewers using the Cochrane Risk of Bias 2.0 (RoB 2) tool [9,10]. We evaluated five domains: bias from the randomization process, deviations from intended interventions, missing outcome data, outcome measurement, and selection of reported results. Each domain was rated as "low risk", "some concerns", or "high risk" based on Cochrane criteria. These assessments were used to describe the overall quality of the included studies but were not factored into the statistical weighting of effect estimates.

Data Synthesis and Statistical Analysis

All statistical analyses were conducted using the R software (Version 4.5.0; R Foundation for Statistical Computing, Vienna, Austria). We performed a frequentist random-effects network meta-analysis using the netmeta package, which maintains the randomization structure of multi-arm trials and incorporates both direct and indirect comparisons, along with heterogeneity within and between studies [11]. Treatment nodes included tirzepatide at 5 mg, tirzepatide at 10 mg, tirzepatide at 15 mg, tirzepatide MTD, semaglutide 2.4 mg, semaglutide MTD, and placebo. The MTDs for tirzepatide and semaglutide were modeled separately from their fixed-dose regimens. Most studies included a placebo as a common comparator, although the SURMOUNT-5 trial directly compared tirzepatide MTD with semaglutide MTD.

The primary outcome was the mean difference (MD) in percent body weight change from baseline. Secondary continuous outcomes included MD in waist circumference. Binary outcomes included the odds of achieving at least 5%, 10%, and 15% weight loss, as well as the odds of experiencing treatment-emergent adverse events, specifically nausea, vomiting, diarrhea, and treatment discontinuation due to adverse events. Continuous outcomes were analyzed using inverse-variance methods, and binary outcomes were analyzed using the Mantel-Haenszel method, both within a random-effects network meta-analysis framework. Results were reported as odds ratios (ORs) with 95% confidence intervals (CI). For binary outcomes with zero-event cells, a continuity correction of 0.5 was applied. We assessed the certainty of evidence for the clinically relevant ≥15% weight loss threshold using the Grading of Recommendations Assessment, Development, and Evaluation (GRADE) framework applied to our network meta-analysis (NMA) estimates.

We assessed transitivity by comparing key clinical and methodological characteristics across treatment comparisons, including mean age, baseline BMI, geographic location, and study duration. Consistency between direct and indirect evidence was evaluated using the design-by-treatment interaction model and local node-splitting methods. Between-study heterogeneity was assessed using the τ² and I² statistics, along with Q tests to evaluate global and within-design inconsistency.

To rank treatments, we calculated surface under the cumulative ranking curve (SUCRA) values for both continuous and binary efficacy outcomes [12]. SUCRA rankings and visualizations were generated entirely in R. Adverse event outcomes were excluded from SUCRA analysis.

Sensitivity Analysis and Assessment of Small-Study Effects

To evaluate the robustness of our findings, a fixed-effects network meta-analysis was performed as a sensitivity analysis. This model assumes a common treatment effect across studies and does not account for between-study heterogeneity. The analysis used the same treatment nodes and effect size metrics as the primary random-effects model. We compared treatment estimates and rankings from both models to assess consistency.

We examined potential small-study effects using a comparison-adjusted funnel plot for the primary outcome of percent change in body weight from baseline. Treatment effect estimates were centered within each comparison group to adjust for differences in baseline comparators across the network. These centered estimates were plotted against their standard errors, and visual symmetry was assessed using overlaid confidence contours at the 90%, 95%, and 99% levels. In addition, we performed a comparison-adjusted Egger-type regression, where the centered treatment effects were regressed on their standard errors. A statistically significant non-zero slope was interpreted as evidence of small-study effects or possible publication bias.

Results

Study Selection

**Figure 1:**
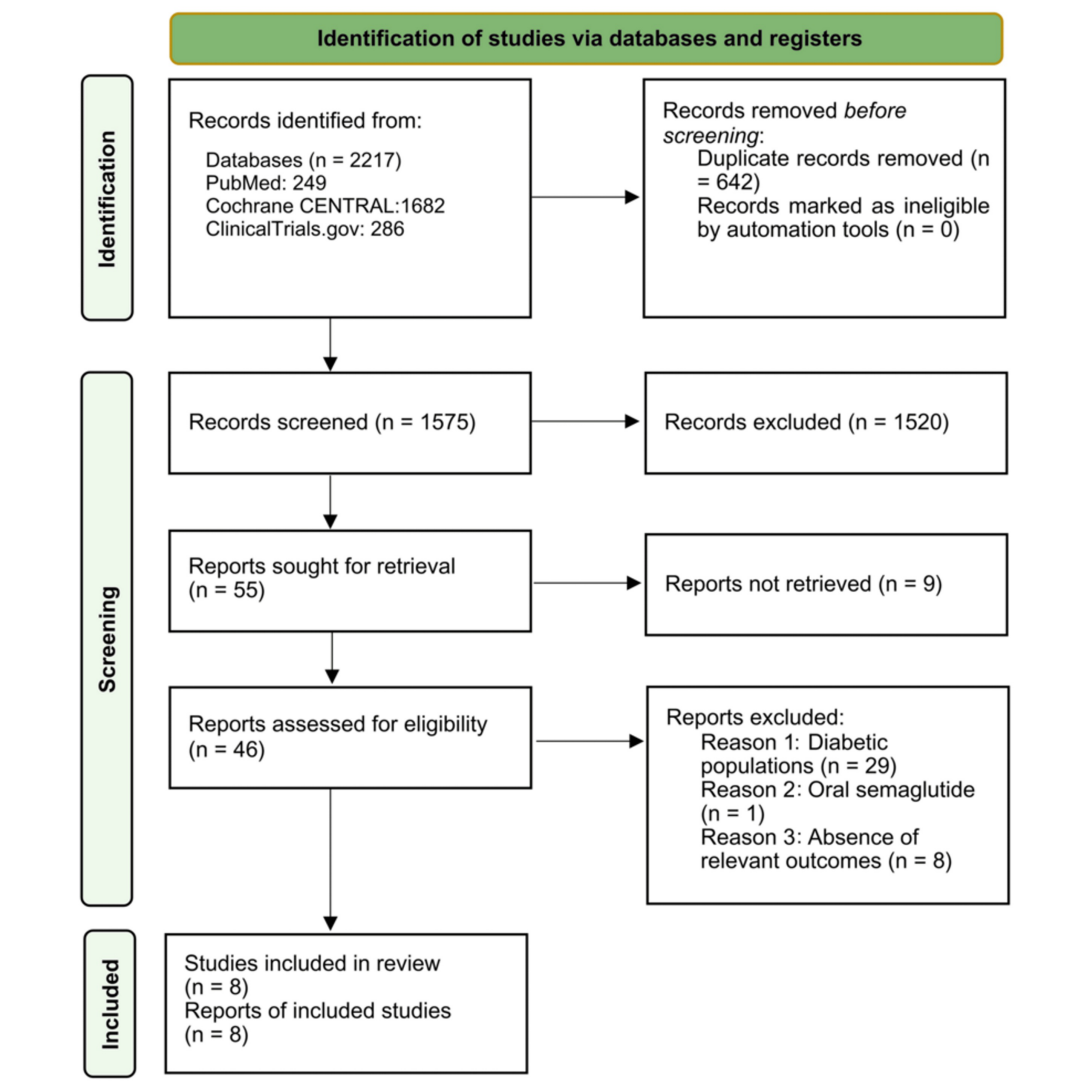
PRISMA flow diagram of study selection PRISMA: Preferred Reporting Items for Systematic Reviews and Meta-Analyses; CENTRAL: Central Register of Controlled Trials

We identified 2,217 records through electronic database searches, including 249 from PubMed, 1,682 from the Cochrane Central Register of Controlled Trials (CENTRAL), and 285 from ClinicalTrials.gov. One additional study was found through manual reference screening. After removing duplicates, 1,575 unique records remained for title and abstract screening. Of these, 46 full-text articles were reviewed for eligibility. Following full-text assessment, 38 articles were excluded for the following reasons: 29 included diabetic populations, one investigated a non-eligible intervention (oral semaglutide), and eight did not report relevant outcomes. Ultimately, eight RCTs [13-20] met all inclusion criteria and were included in the final network meta-analysis. The study selection process is summarized in the PRISMA flow diagram (Figure 1).

Study Characteristics

A total of eight RCTs, including 7,179 participants, were included in the analysis. Three trials evaluated injectable semaglutide 2.4 mg (STEP-1, STEP-3, and STEP-5), while five trials assessed subcutaneous tirzepatide at fixed doses of 5 mg, 10 mg, 15 mg, or using a stepwise MTD approach (SURMOUNT-1, SURMOUNT-3, SURMOUNT-5, SURMOUNT-CN, and SURMOUNT-J). The SURMOUNT-5 trial also included a comparator arm using semaglutide escalated to its MTD (1.7 or 2.4 mg). All studies were multicenter RCTs conducted over durations ranging from 52 to 104 weeks. Except for SURMOUNT-5, which was open-label due to differences in the labeling of the injection device, all trials were double-blind.

Across studies, the mean participant age ranged from 34.7 to 52.3 years, and more than 65% of participants in each trial were women. The baseline body weight ranged from 91.3 to 113.4 kg, and the BMI ranged from 32 to 39.4 kg/m². All trials reported a percentage change in body weight from baseline as the primary outcome. The semaglutide trials were sponsored by Novo Nordisk, while the tirzepatide trials, including SURMOUNT-5, were funded by Eli Lilly and Company. All included studies were registered on ClinicalTrials.gov and received appropriate ethical approval. Detailed characteristics of the included trials are provided in Table 1.

**Table 1:** Characteristics of the included RCTs RCT: randomized controlled trial; T: treatment; C: comparator; BMI: body mass index; M: male; F: female; MTD: maximum tolerated dose; STEP: Semaglutide Treatment Effect in People with obesity; SURMOUNT: SURpass tirzepatide Obesity Multi-dose evalUaTion trial Sources: [13-20]

Author (year)	Study design	Trial name	Treatment	Sample size (T/C)	Comparator	Mean age (T/C)	Sex (M/F) (T/C)	Baseline weight (T/C)	Baseline BMI (T/C)	Primary outcome	Follow-up	Blinding	Funding	Trial registration
Wilding et al. (2021) [13]	RCT	STEP-1	Semaglutide 2.4 mg	1306/655	Placebo	46 (13)/47 (12)	351/973; 157/498	105.4 (22.1)/105.2 (21.5)	37.8 (6.7)/38.0 (6.5)	Weight loss	68 weeks	Double-blind	Novo Nordisk	NCT03548935
Wadden et al. (2021) [14]	RCT	STEP-3	Semaglutide 2.4 mg	407/204	Placebo	46 (13)/46 (13)	92/315; 24/180	106.9 (22.8)/103.7 (22.9)	38.1 (6.7)/37.8 (6.9)	Weight loss	68 weeks	Double-blind	Novo Nordisk	NCT03611582
Garvey et al. (2022) [15]	RCT	STEP-5	Semaglutide 2.4 mg	152/152	Placebo	47.3 (11.7)/47.4 (10.3)	29/123; 39/113	105.6 (20.8)/106.5 (23.1)	38.6 (6.7)/38.5 (7.2)	Weight loss	104 weeks	Double-blind	Novo Nordisk	NCT03693430
Jastreboff et al. (2022) [16]	RCT	SURMOUNT-1	Tirzepatide 5 mg	630/643	Placebo	45.6 (12.7)/44.4 (12.5)	206/426; 207/436	102.9 (20.7)/104.8 (21.4)	37.4 (6.6)/38.2 (6.9)	Weight loss	72 weeks	Double-blind	Eli Lilly and Company	NCT04184622
			Tirzepatide 10 mg	636/643		44.7 (12.4)/44.4 (12.5)	209/427; 207/436	105.8 (23.3)/104.8 (21.4)	38.2 (7.0)/38.2 (6.9)					
			Tirzepatide 15 mg	630/643		44.9 (12.3)/44.4 (12.5)	205/425; 207/436	105.6 (22.9)/104.8 (21.4)	38.1 (6.7)/38.2 (6.9)					
Wadden et al. (2023) [17]	RCT	SURMOUNT-3	Tirzepatide 10-15 mg (MTD)	287/292	Placebo	45.4 (12.6)/45.7 (11.8)	106/181; 109/183	102.5 (22.1)/101.3 (20.7)	36.1 (6.1)/35.7 (6.4)	Weight loss	72 weeks	Double-blind	Eli Lilly and Company	NCT04657016
Zhao et al. (2024) [18]	RCT	SURMOUNT-CN	Tirzepatide 10 mg	70/69	Placebo	34.7 (7.2)/37.8 (10.2)	35/35; 36/33	92.2 (16.2)/92.0 (15.8)	32.6 (4.1)/32.4 (3.6)	Weight loss	52 weeks	Double-blind	Eli Lilly and Company	NCT05024032
			Tirzepatide 15 mg	71/69		35.8 (9.3)/37.8 (10.2)	36/35; 36/33	91.3 (16.2)/92.0 (15.8)	32.0 (3.7)/32.4 (3.6)					
Kadowaki et al. (2025) [19]	RCT	SURMOUNT-J	Tirzepatide 10 mg	73/75	Placebo	49 (10.9)/52.3 (10.9)	43/30; 45/30	92.4 (15.0)/92.0 (15.3)	33.2 (4.1)/33.7 (4.9)	Weight loss	72 weeks	Double-blind	Eli Lilly and Company	NCT04844918
			Tirzepatide 15 mg	77/75		51.1 (10.3)/52.3 (10.9)	45/32; 45/30	91.7 (14.8)/92.0 (15.3)	33.6 (4.3)/33.7 (4.9)					
Aronne et al. (2025) [20]	RCT	SURMOUNT-5	Tirzepatide 10-15 mg (MTD)	374/376	Semaglutide 1.7-2.4 mg (MTD)	45 (12.9)/44.4 (12.7)	132/242; 133/243	112.7 (24.8)/113.4 (26.3)	39.4 (7.4)/39.4 (7.7)	Weight loss	72 weeks	Open-label	Eli Lilly and Company	NCT05822830

Risk of Bias Assessment

**Figure 2:**
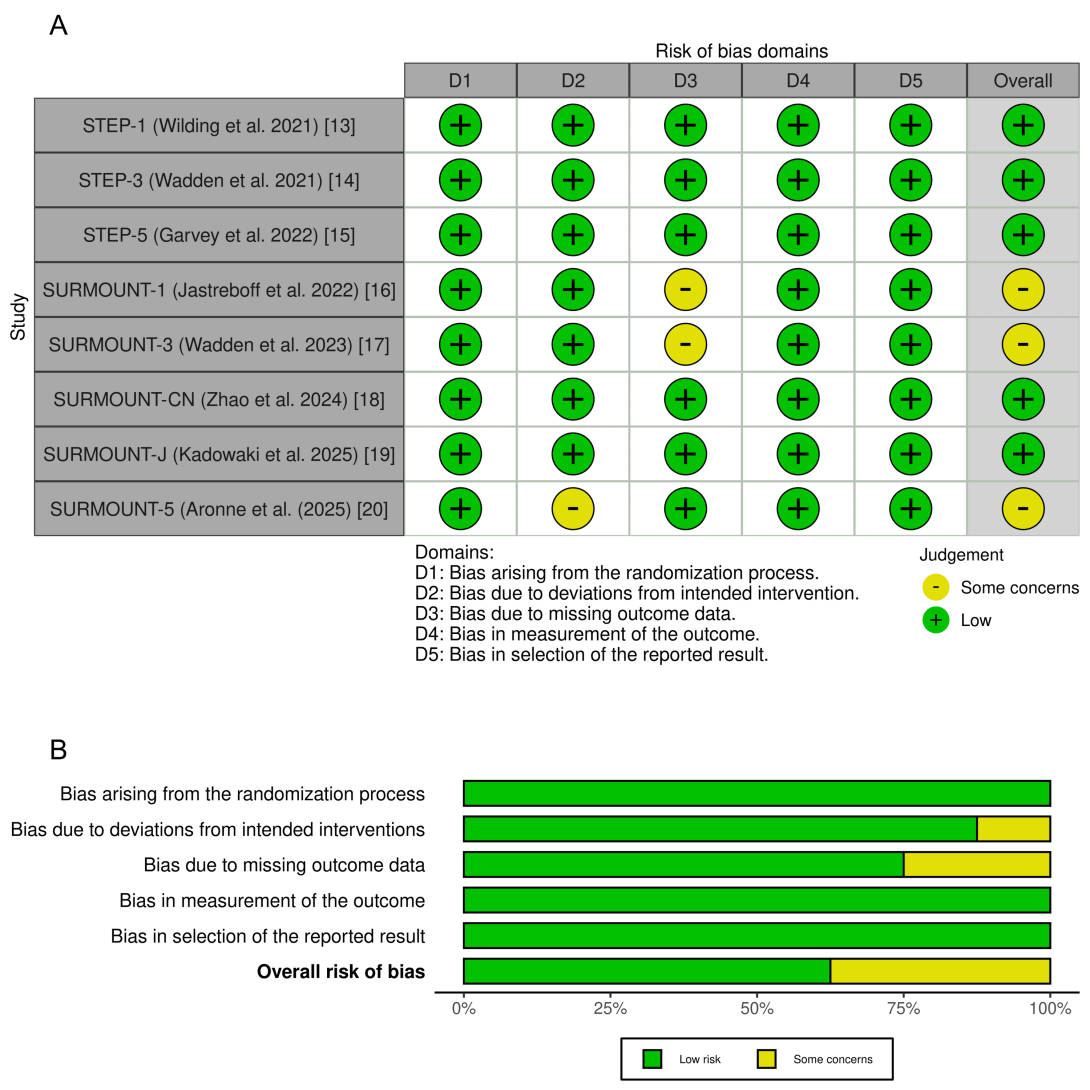
(A) Risk of bias summary across all included trials. (B) Risk of bias judgments by individual trial RoB 2: Risk of Bias 2.0; STEP: Semaglutide Treatment Effect in People with obesity; SURMOUNT: SURpass tirzepatide Obesity Multi-dose evalUaTion trial; SURMOUNT-CN: SURMOUNT China; SURMOUNT-J: SURMOUNT Japan Sources: [13-20]

Risk of bias was evaluated using the Cochrane Risk of Bias 2.0 (RoB 2) tool across five domains. As shown in Figure 2A, five of the eight trials were rated as having a low risk of bias in all domains. These included the semaglutide trials (STEP-1, STEP-3, and STEP-5) and two tirzepatide trials (SURMOUNT-CN and SURMOUNT-J). The remaining three trials, SURMOUNT-1 [16], SURMOUNT-3 [17], and SURMOUNT-5 [20], were assessed as having "some concerns" in one domain each. For SURMOUNT-1 and SURMOUNT-3, this was due to missing outcome data (D3). For SURMOUNT-5, concerns were noted in the domain related to deviations from intended interventions (D2), primarily due to its open-label design. These concerns were judged to be minor and unlikely to meaningfully influence the overall risk of bias. Figure 2B presents a summary of domain-level risk across all trials, highlighting the overall strong methodological quality of the included studies.

Network Geometry 

**Figure 3:**
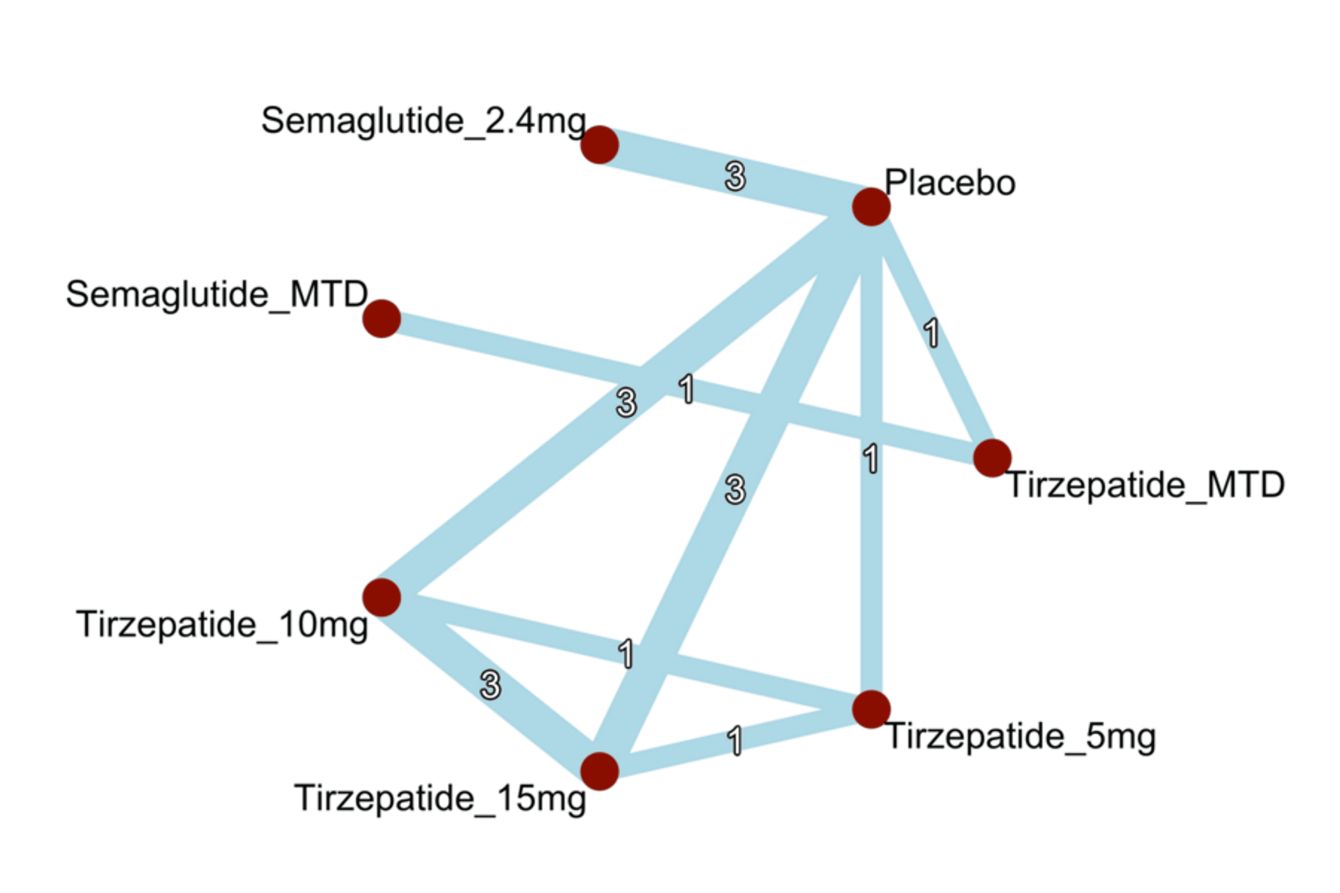
Network geometry of the included interventions MTD: maximum tolerated dose Sources: [13-20]

Figure 3 depicts the geometry of the treatment network for percentage body weight change. Most interventions were linked either directly or indirectly through a common placebo comparator, forming a predominantly star-shaped network. Several active treatments, particularly tirzepatide 5 mg, 10 mg, and 15 mg, were directly compared within multi-arm trials. Tirzepatide MTD was evaluated both against placebo and in a head-to-head comparison with semaglutide MTD in the SURMOUNT-5 trial. No direct comparisons were observed between semaglutide 2.4 mg and any tirzepatide dose. These limitations emphasize the importance of indirect evidence in estimating treatment contrasts. In the network plot, edge thickness represents the number of studies contributing to each treatment comparison, with placebo emerging as the most commonly connected node. Overall, the network was sufficiently connected to support reliable mixed-treatment comparisons among all included interventions.

Percentage Body Weight Change

**Figure 4:**
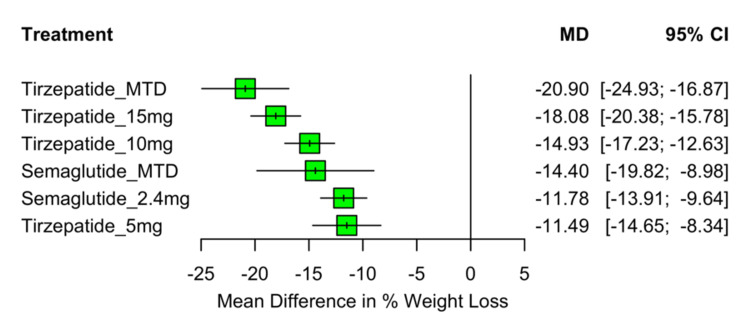
Forest plot of mean percentage body weight change from baseline MD: mean difference; CI: confidence interval; MTD: maximum tolerated dose Sources: [13-20]

A total of eight RCTs involving 7,179 participants were included in the network meta-analysis, evaluating the mean percentage change in body weight from baseline. All active treatments resulted in significantly greater weight loss compared to the placebo. Tirzepatide at its MTD showed the greatest effect, with an MD of −20.90% (95% CI: −24.93 to −16.87), followed by tirzepatide 15 mg (MD: −18.08%; 95% CI: −20.38 to −15.78), tirzepatide 10 mg (MD: −14.93%; 95% CI: −17.23 to −12.63), semaglutide MTD (MD: −14.40%; 95% CI: −19.82 to −8.98), semaglutide 2.4 mg (MD: −11.78%; 95% CI: −13.91 to −9.64), and tirzepatide 5 mg (MD: −11.49%; 95% CI: −14.65 to −8.34). All comparisons with placebo were statistically significant (p<0.0001). A summary of the relative treatment effects is presented in the forest plot in Figure 4.

We observed substantial heterogeneity (τ²=2.73; I²=73%, 95% CI: 41.9% to 87.4%), which is expected in studies involving behavioral components and diverse populations. Overall inconsistency across the network was statistically significant (Q=22.21; df=6; p=0.0011), driven mainly by heterogeneity within study designs (Q=15.12; df=4; p=0.0045). However, there was also significant between-design inconsistency (Q=7.09; df=2; p=0.0288), suggesting some disagreement between direct and indirect estimates, despite most trials sharing a placebo comparator. Node-splitting and local inconsistency analyses were not performed due to the limited number of closed loops in the network, which restricted their interpretability.

**Figure 5:**
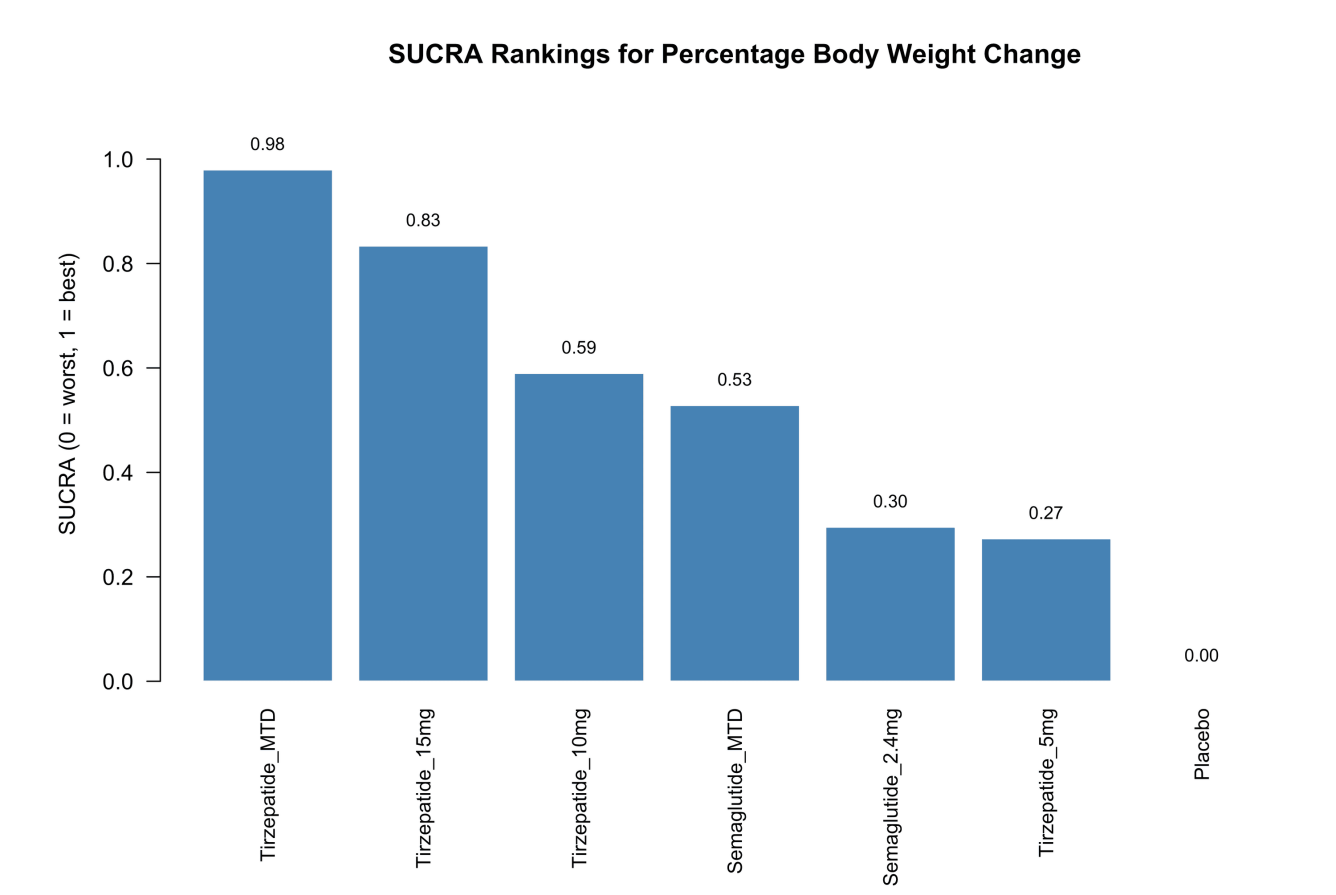
SUCRA rankings of interventions for percentage body weight change SUCRA: surface under the cumulative ranking curve; MTD: maximum tolerated dose Sources: [13-20]

Treatment rankings based on SUCRA values supported the point estimates. Tirzepatide MTD had the highest probability of being the most effective treatment for weight reduction (SUCRA=0.98), followed by tirzepatide 15 mg (0.83), tirzepatide 10 mg (0.59), semaglutide MTD (0.53), semaglutide 2.4 mg (0.30), and tirzepatide 5 mg (0.27). Placebo ranked lowest (SUCRA=0.00), as expected. These rankings, shown in Figure 5, are consistent with the effect estimates from the meta-analysis and further reinforce the robustness of the comparative efficacy findings.

Waist Circumference Reduction

**Figure 6:**
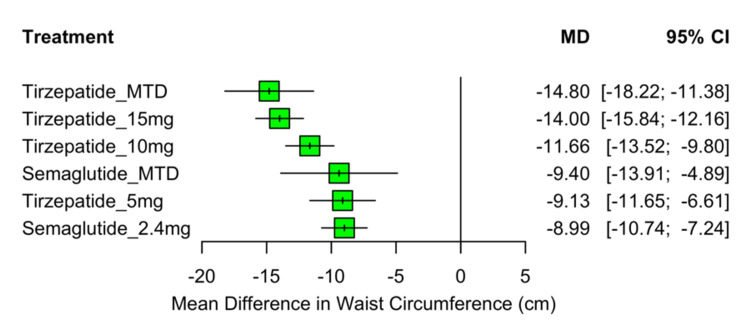
Forest plot of mean change in waist circumference from baseline MD: mean difference; CI: confidence interval; MTD: maximum tolerated dose Sources: [13-20]

All eight RCTs, including 7,179 participants, reported changes in waist circumference from baseline. All active treatments resulted in statistically significant reductions compared to the placebo (p<0.0001 for all comparisons). The largest reduction was observed with tirzepatide at its MTD, with a mean difference of −14.80 cm (95% CI: −18.22 to −11.38), followed by tirzepatide 15 mg (MD: −14.00 cm; 95% CI: −15.84 to −12.16), tirzepatide 10 mg (MD: −11.66 cm; 95% CI: −13.52 to −9.80), semaglutide MTD (MD: −9.40 cm; 95% CI: −13.91 to −4.89), tirzepatide 5 mg (MD: −9.13 cm; 95% CI: −11.65 to −6.61), and semaglutide 2.4 mg (MD: −8.99 cm; 95% CI: −10.74 to −7.24). These results are presented in the forest plot shown in Figure 6, highlighting the greater efficacy of higher-dose tirzepatide regimens in reducing central adiposity.

Moderate heterogeneity was observed across studies (τ²=1.56; I²=62.4%; 95% CI: 14.5% to 83.5%), which may reflect differences in baseline waist circumference or variations in concurrent lifestyle interventions. Within-design inconsistency was not statistically significant (Q=5.52; df=4; p=0.2381), indicating coherence among comparisons within individual study designs. However, between-design inconsistency was statistically significant (Q=10.44; df=2; p=0.0054), suggesting some disagreement between direct and indirect comparisons. Due to the limited number of closed loops beyond the shared placebo comparator, local inconsistency analyses (e.g., node-splitting) were not conducted.

**Figure 7:**
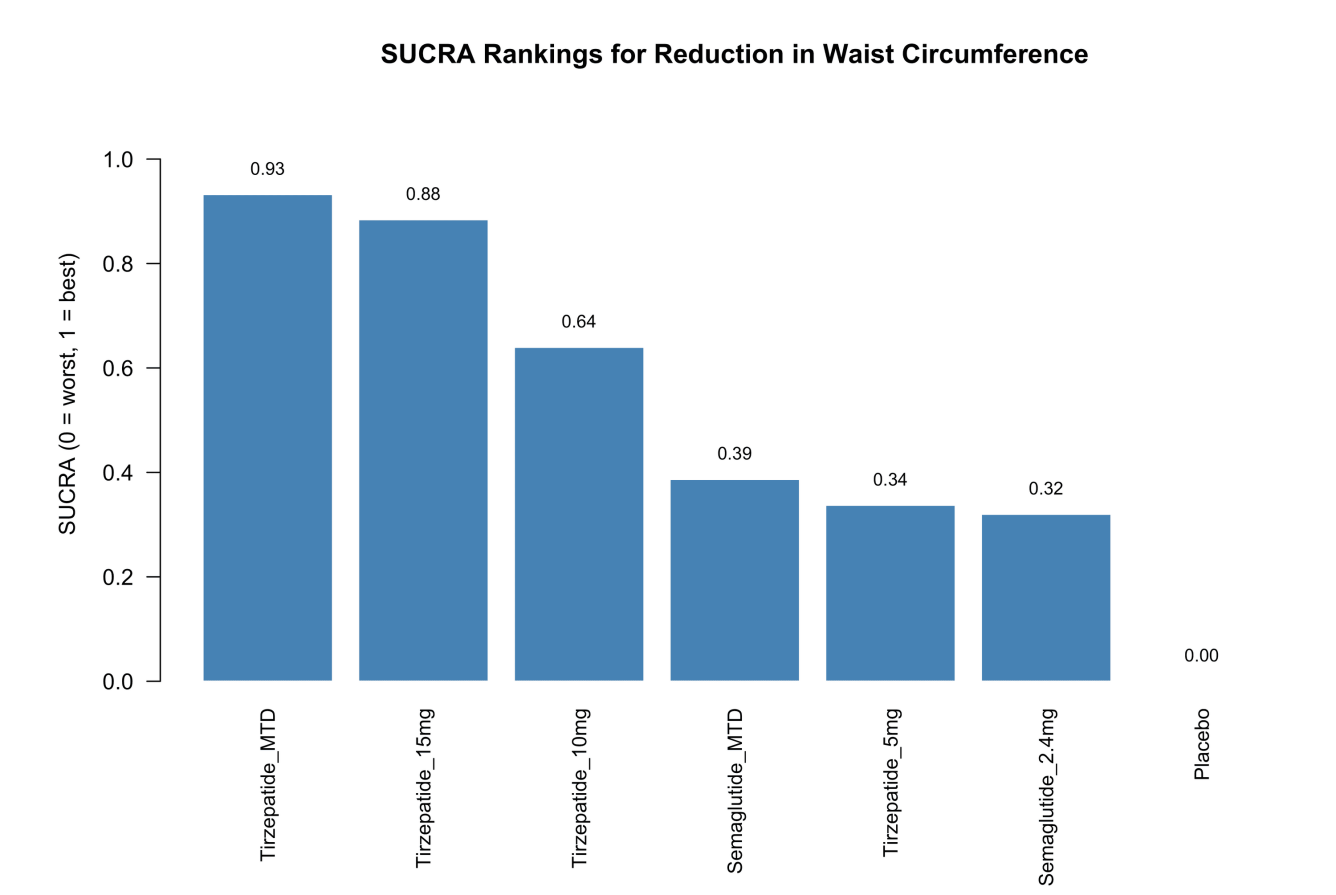
SUCRA rankings of interventions for reduction in waist circumference SUCRA: surface under the cumulative ranking curve; MTD: maximum tolerated dose Sources: [13-20]

Treatment rankings based on SUCRA values are presented in Figure 7. Tirzepatide MTD had the highest likelihood of being the most effective treatment for waist circumference reduction (SUCRA=0.93), followed by tirzepatide 15 mg (SUCRA=0.88) and tirzepatide 10 mg (SUCRA=0.64). Semaglutide MTD had a moderate ranking (0.39), while tirzepatide 5 mg (0.34) and semaglutide 2.4 mg (0.32) ranked similarly lower. Placebo had the lowest SUCRA value of 0.00. These rankings align with the observed treatment effects and support the relative advantage of higher-dose tirzepatide in targeting central fat reduction.

Achievement of Clinically Meaningful Weight Loss (≥5%, ≥10%, ≥15%)

**Figure 8:**
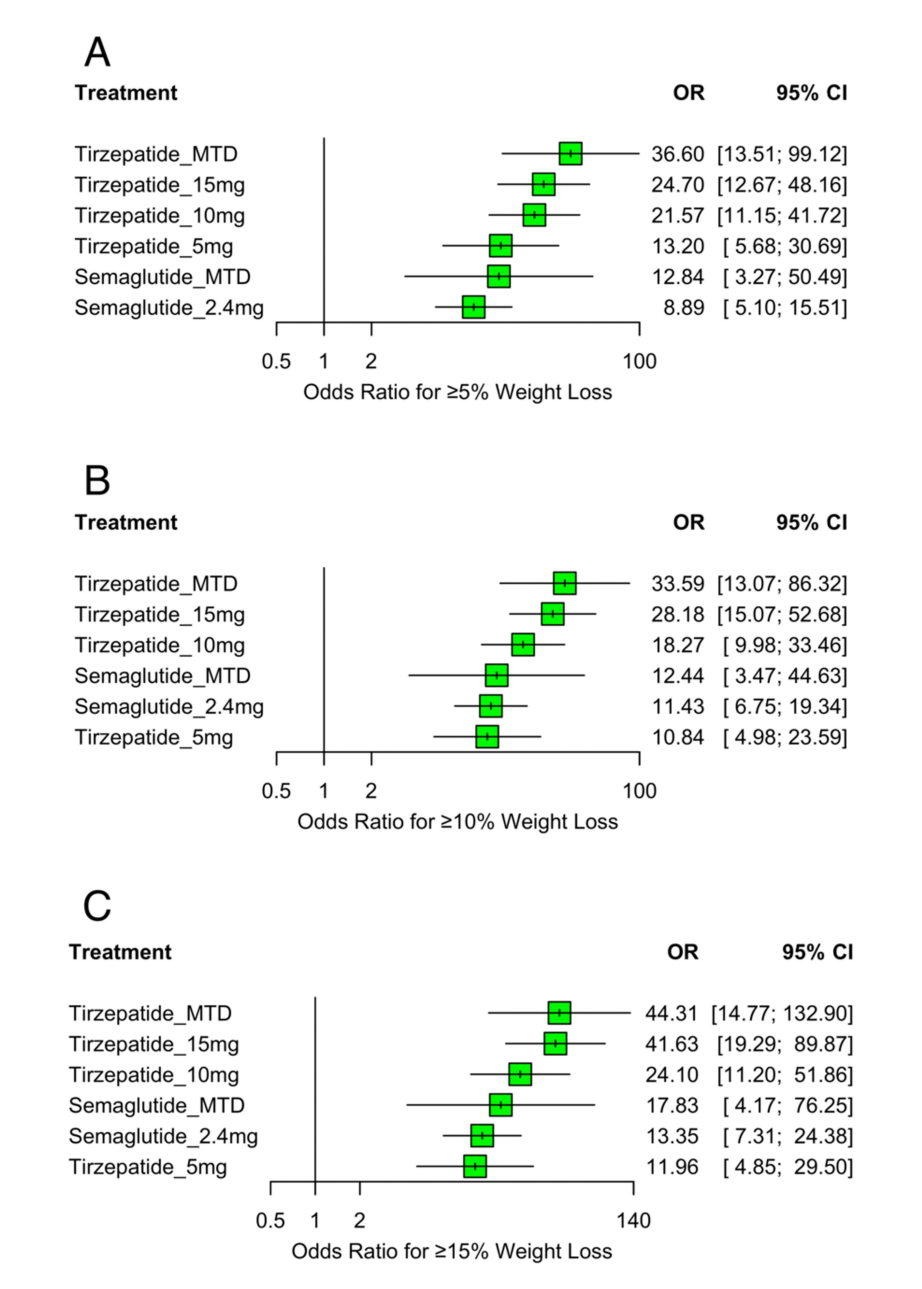
Forest plot of OR for achieving ≥5%, ≥10%, and ≥15% weight loss. (A) shows OR and 95% CI for achieving ≥5% body weight loss, (B) shows results for ≥10% weight loss, and (C) shows results for ≥15% weight loss OR: odds ratio; CI: confidence interval; MTD: maximum tolerated dose Sources: [13-20]

All eight RCTs, comprising 7,179 participants, reported outcomes for the proportion of individuals achieving clinically meaningful weight loss at three thresholds, ≥5%, ≥10%, and ≥15%, of baseline body weight. Across all thresholds, every active treatment demonstrated significantly higher odds of achieving weight loss compared to placebo (p<0.0001 for all comparisons). The forest plots for these comparisons are presented in Figure 8A-8C.

At the ≥5% threshold, tirzepatide at its MTD had the highest effect (OR: 36.60; 95% CI: 13.51 to 99.12), followed by tirzepatide 15 mg (OR: 24.70; 95% CI: 12.67 to 48.16), 10 mg (OR: 21.57; 95% CI: 11.15 to 41.72), and 5 mg (OR: 13.20; 95% CI: 5.68 to 30.69). Semaglutide MTD (OR: 12.84; 95% CI: 3.27 to 50.49) and semaglutide 2.4 mg (OR: 8.89; 95% CI: 5.10 to 15.51) followed.

For the ≥10% threshold, tirzepatide MTD again ranked highest (OR: 33.59; 95% CI: 13.07 to 86.32), followed by tirzepatide 15 mg (OR: 28.18; 95% CI: 15.07 to 52.68), tirzepatide 10 mg (OR: 18.27; 95% CI: 9.98 to 33.46), semaglutide MTD (OR: 12.44; 95% CI: 3.47 to 44.63), semaglutide 2.4 mg (OR: 11.43; 95% CI: 6.75 to 19.34), and tirzepatide 5 mg (OR: 10.84; 95% CI: 4.98 to 23.59).

At the highest threshold, ≥15%, the same ranking pattern persisted. Tirzepatide MTD showed the greatest odds (OR: 44.31; 95% CI: 14.77 to 132.90), followed closely by tirzepatide 15 mg (OR: 41.63; 95% CI: 19.29 to 89.87), tirzepatide 10 mg (OR: 24.10; 95% CI: 11.20 to 51.86), semaglutide MTD (OR: 17.83; 95% CI: 4.17 to 76.25), semaglutide 2.4 mg (OR: 13.35; 95% CI: 7.31 to 24.38), and tirzepatide 5 mg (OR: 11.96; 95% CI: 4.85 to 29.50).

Moderate heterogeneity was observed across all response thresholds (I²=68.5% to 73.2%). Inconsistency was minimal for the ≥5% and ≥10% thresholds, but statistically significant between-design inconsistency was noted at the ≥15% threshold (Q=8.06; df=2; p=0.0177), possibly reflecting the greater reliance on indirect evidence and lower event rates at higher weight loss levels.

**Figure 9:**
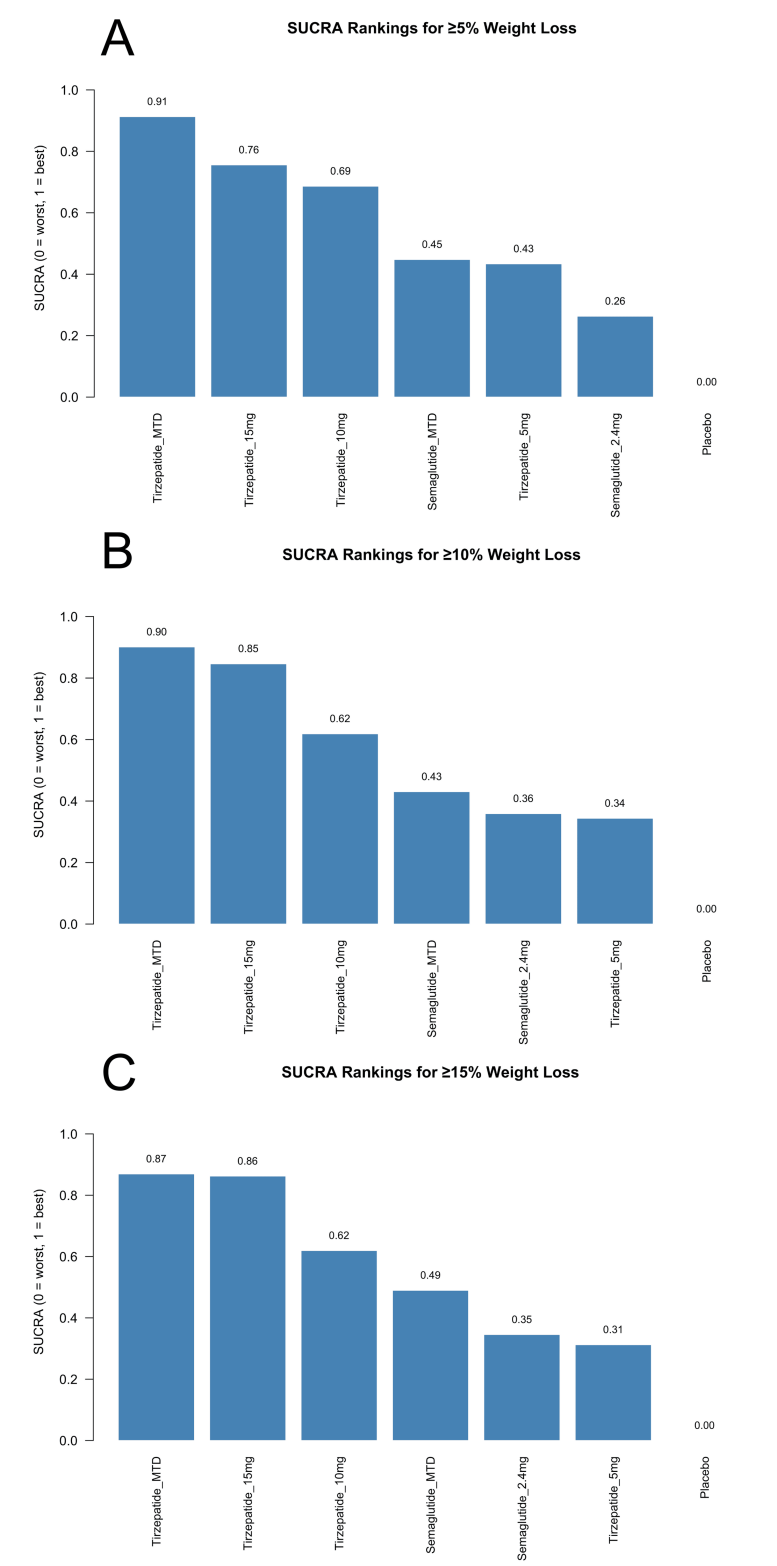
SUCRA rankings of interventions for achieving (A) ≥5% body weight loss, (B) ≥10% body weight loss, and (C) ≥15% body weight loss SUCRA: surface under the cumulative ranking curve; MTD: maximum tolerated dose Sources: [13-20]

SUCRA rankings (Figure 9A-9C) showed a consistent dose-dependent pattern, with higher doses of tirzepatide ranking above semaglutide at all thresholds. For the ≥5% outcome, tirzepatide MTD ranked highest (SUCRA=0.91), followed by tirzepatide 15 mg (0.76), tirzepatide 10 mg (0.69), semaglutide MTD (0.45), tirzepatide 5 mg (0.43), and semaglutide 2.4 mg (0.26). At the ≥10% threshold, tirzepatide MTD (0.90) and tirzepatide 15 mg (0.85) remained top-ranked, followed by tirzepatide 10 mg (0.62), semaglutide MTD (0.43), semaglutide 2.4 mg (0.36), and tirzepatide 5 mg (0.34). A similar pattern was observed at the ≥15% threshold, with tirzepatide MTD (0.87) and tirzepatide 15 mg (0.86) leading, followed by tirzepatide 10 mg (0.62), semaglutide MTD (0.49), semaglutide 2.4 mg (0.35), and tirzepatide 5 mg (0.31). In all cases, the placebo ranked lowest (SUCRA=0.00). These rankings aligned with the treatment effect estimates from the forest plots and demonstrated a clear dose-response relationship, with higher-dose tirzepatide consistently outperforming semaglutide across all weight loss thresholds.

Sensitivity Analysis: Fixed-Effects Model

To evaluate the strength of the primary findings, we performed a sensitivity analysis using a fixed-effects network meta-analysis. This model assumes a common treatment effect across all studies and does not account for between-study heterogeneity. The direction and relative ranking of treatment effects remained consistent with those observed under the random-effects model. Tirzepatide at its MTD continued to demonstrate the greatest reduction in percentage body weight (MD: −20.90%; 95% CI: −23.29 to −18.51), followed by tirzepatide 15 mg (MD: −18.00%; 95% CI: −19.19 to −16.81), tirzepatide 10 mg (MD: −15.60%; 95% CI: −16.79 to −14.40), semaglutide MTD (MD: −14.40%; 95% CI: −17.29 to −11.51), semaglutide 2.4 mg (MD: −12.11%; 95% CI: −12.83 to −11.39), and tirzepatide 5 mg (MD: −11.67%; 95% CI: −12.98 to −10.36). These results confirm that the primary conclusions are robust and not sensitive to the choice of modeling assumptions regarding heterogeneity.

Assessment of Small-Study Effects

**Figure 10:**
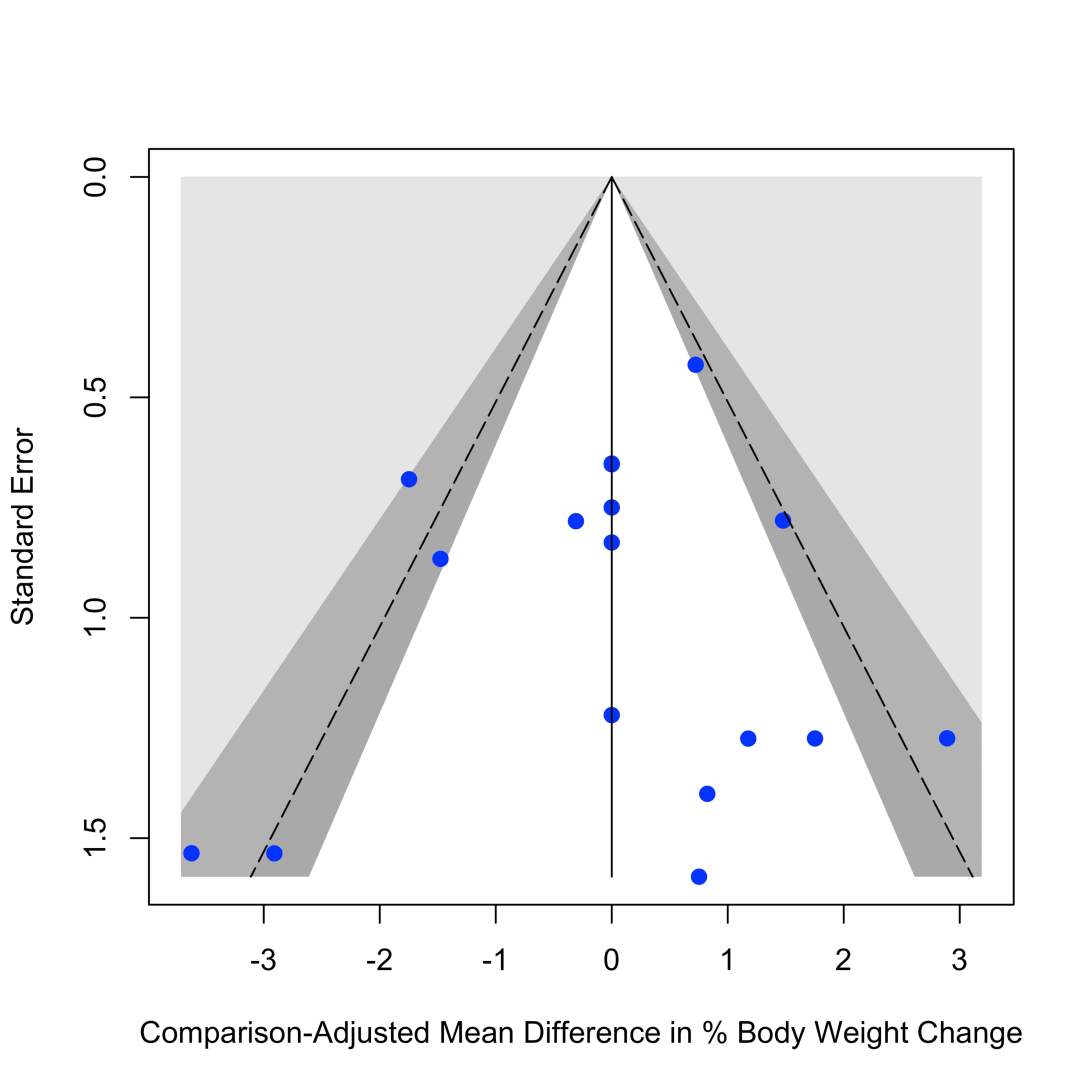
Comparison-adjusted funnel plot for mean percentage body weight change Sources: [13-20]

The comparison-adjusted funnel plot (Figure 10) showed a generally symmetrical distribution of study comparisons centered around the network mean. Of the 17 pairwise comparisons, 11 (64.7%) were located within the 90% confidence region, two (11.8%) between the 90% and 95% region, three (17.6%) between the 95% and 99% region, and one (5.9%) beyond the 99% confidence region. This distribution did not suggest evidence of asymmetry. Consistent with the visual assessment, the comparison-adjusted Egger-type regression yielded a slope of 1.63 (p=0.146), indicating no statistically significant association between effect size and standard error. Taken together, these results do not indicate the presence of small-study effects or publication bias within the network.

Discontinuation Due to Adverse Events

**Figure 11:**
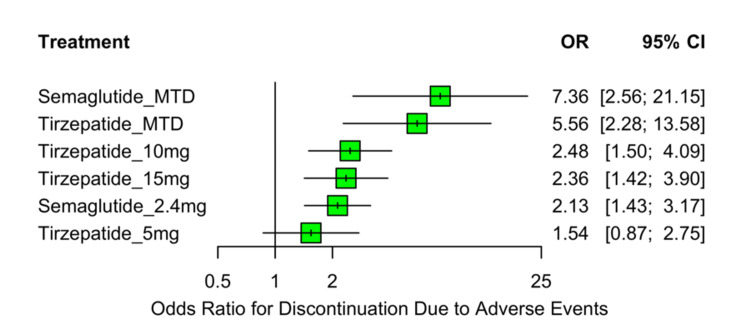
Forest plot of OR for discontinuation due to adverse events OR: odds ratio; CI: confidence interval; MTD: maximum tolerated dose Sources: [13-20]

All active treatments, except for tirzepatide 5 mg, were associated with a statistically significant increase in the odds of treatment discontinuation due to adverse events compared to placebo (Figure 11). The highest discontinuation rates were observed with semaglutide MTD (OR: 7.36; 95% CI: 2.56 to 21.15; p=0.0002) and tirzepatide MTD (OR: 5.56; 95% CI: 2.28 to 13.58; p=0.0002). Elevated odds were also seen with tirzepatide 10 mg (OR: 2.48; 95% CI: 1.50 to 4.09; p=0.0004), tirzepatide 15 mg (OR: 2.36; 95% CI: 1.42 to 3.90; p=0.0009), and semaglutide 2.4 mg (OR: 2.13; 95% CI: 1.43 to 3.17; p=0.0002). Tirzepatide 5 mg did not differ significantly from placebo (OR: 1.54; 95% CI: 0.87 to 2.75; p=0.1419). No heterogeneity was detected across studies (τ²=0; I²=0%), and there was no evidence of inconsistency between direct and indirect comparisons (Q=3.69; df=6; p=0.7185), suggesting stability and coherence in the pooled estimates.

Gastrointestinal Adverse Events

**Figure 12:**
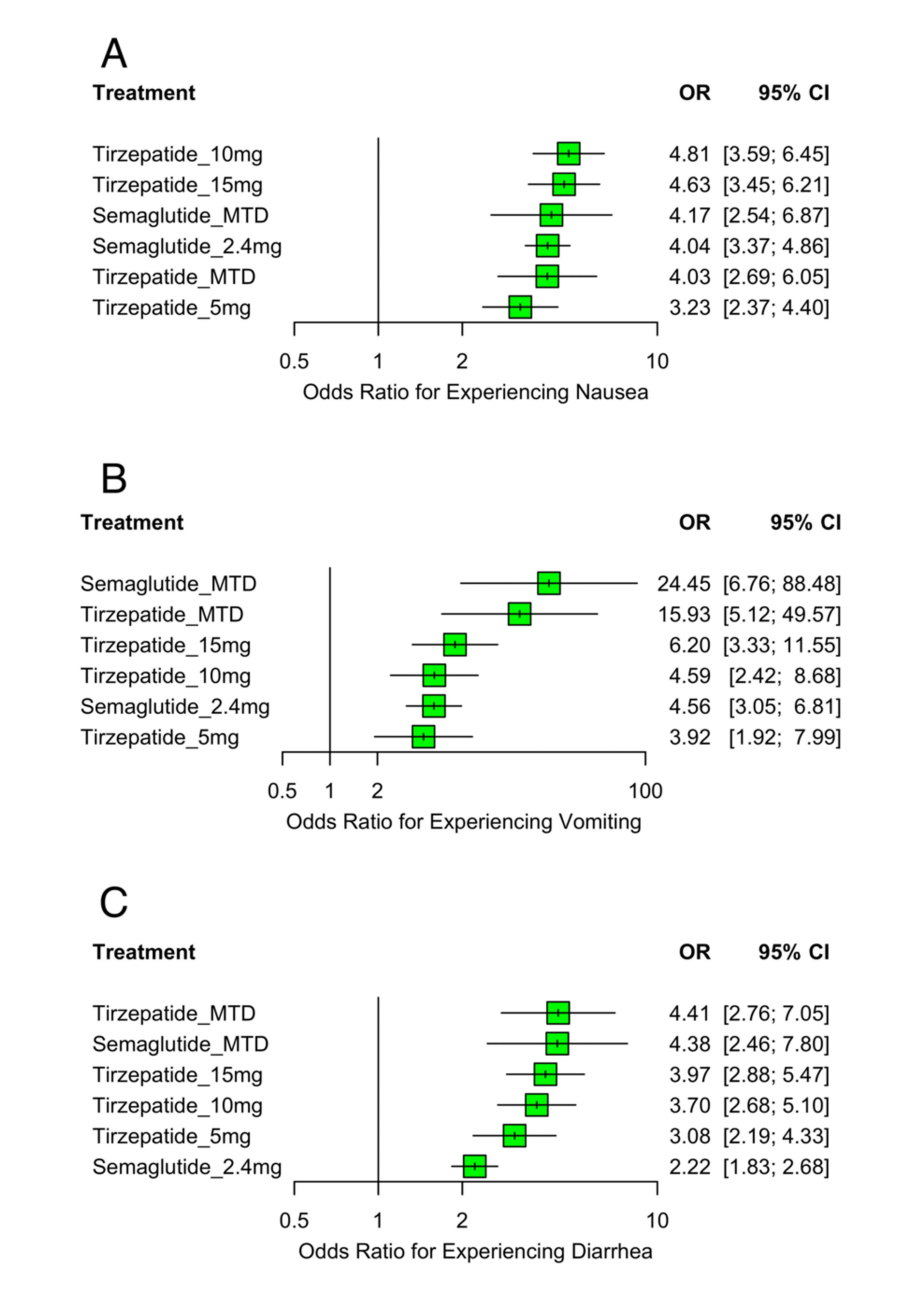
Forest plots of OR for (A) nausea, (B) vomiting, and (C) diarrhea OR: odds ratio; CI: confidence interval; MTD: maximum tolerated dose Sources: [13-20]

All active treatments were associated with significantly increased odds of gastrointestinal adverse events compared to placebo. Figure 12 summarizes the findings for three common gastrointestinal outcomes: nausea (Figure 12A), vomiting (Figure 12B), and diarrhea (Figure 12C).

For nausea, the highest odds were observed with tirzepatide 10 mg (OR: 4.81; 95% CI: 3.59 to 6.45), followed by tirzepatide 15 mg (OR: 4.63; 95% CI: 3.45 to 6.21), semaglutide MTD (OR: 4.17; 95% CI: 2.54 to 6.87), semaglutide 2.4 mg (OR: 4.04; 95% CI: 3.37 to 4.86), tirzepatide MTD (OR: 4.03; 95% CI: 2.69 to 6.05), and tirzepatide 5 mg (OR: 3.23; 95% CI: 2.37 to 4.40). Heterogeneity was negligible (I²=0%), and no inconsistency was detected.

For vomiting, semaglutide MTD showed the highest risk (OR: 24.45; 95% CI: 6.76 to 88.48), followed by tirzepatide MTD (OR: 15.93; 95% CI: 5.12 to 49.57), tirzepatide 15 mg (OR: 6.20; 95% CI: 3.33 to 11.55), tirzepatide 10 mg (OR: 4.59; 95% CI: 2.42 to 8.68), semaglutide 2.4 mg (OR: 4.56; 95% CI: 3.05 to 6.81), and tirzepatide 5 mg (OR: 3.92; 95% CI: 1.92 to 7.99). Heterogeneity was low (I²=31.6%), and there was no evidence of inconsistency.

For diarrhea, the highest odds were observed with tirzepatide MTD (OR: 4.41; 95% CI: 2.76 to 7.05), followed closely by semaglutide MTD (OR: 4.38; 95% CI: 2.46 to 7.80), tirzepatide 15 mg (OR: 3.97; 95% CI: 2.88 to 5.47), tirzepatide 10 mg (OR: 3.70; 95% CI: 2.68 to 5.10), tirzepatide 5 mg (OR: 3.08; 95% CI: 2.19 to 4.33), and semaglutide 2.4 mg (OR: 2.22; 95% CI: 1.83 to 2.68). No heterogeneity or inconsistency was detected (I²=0%). These findings highlight a consistent pattern of gastrointestinal side effects associated with both tirzepatide and semaglutide, with a tendency toward greater risk at higher doses. *Certainty of Evidence for ≥15% Weight Loss*

**Figure 13:**
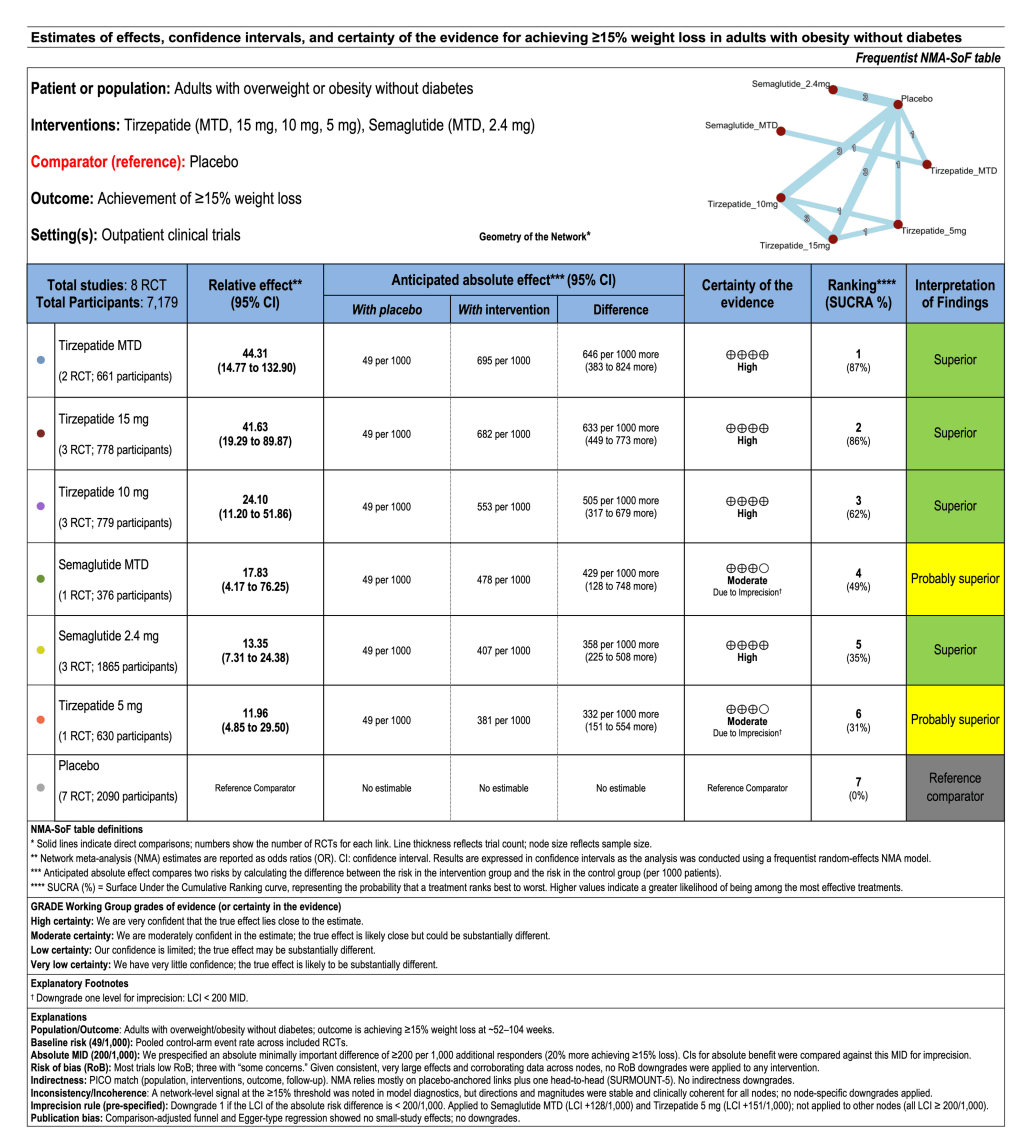
NMA-GRADE summary of findings table for achieving ≥15% weight loss NMA-GRADE: Grading of Recommendations Assessment, Development, and Evaluation for Network Meta-Analysis

Since the ≥15% weight loss threshold is clinically meaningful for obesity management, we applied the GRADE approach to our NMA estimates, following the method described by Yepes-Nuñez et al. [21], to evaluate the certainty of evidence for this outcome. The results are summarized in the NMA-GRADE summary of findings table (Figure 13).

Discussion

Summary of Key Findings

This network meta-analysis assessed the comparative efficacy and tolerability of various doses of tirzepatide and semaglutide in non-diabetic adults with obesity. Tirzepatide at its MTD and at 15 mg consistently ranked highest for percentage body weight reduction, as supported by SUCRA values and sensitivity analyses using both fixed- and random-effects models. A clear dose-response relationship was observed, with greater reductions in body weight and waist circumference at higher tirzepatide doses. These efficacy advantages extended to categorical weight loss outcomes, where higher dose tirzepatide regimens showed the greatest likelihood of achieving ≥5%, ≥10%, and ≥15% body weight reduction. However, this enhanced efficacy came at the cost of lower tolerability. Both tirzepatide MTD and semaglutide MTD were associated with the highest odds of treatment discontinuation due to adverse events. Gastrointestinal side effects, including nausea, vomiting, and diarrhea, were more frequent with all active treatments, particularly at higher doses. No evidence of publication bias or small-study effects was detected based on funnel plot symmetry and Egger-type regression, further supporting the reliability of these findings.

Interpretation and Contextualization

Obesity in non-diabetic adults remains a pressing clinical and public health concern, and there is growing demand for medications that achieve substantial weight loss without compromising tolerability. Our analysis indicates that tirzepatide, particularly at its MTD and at 15 mg, produces greater weight reduction than semaglutide 2.4 mg, semaglutide MTD, or lower tirzepatide doses. The clear dose-response pattern mirrors findings from the SURMOUNT trials, where escalating tirzepatide doses led to progressively larger weight losses [16,19], and aligns with prior meta-analytic evidence demonstrating greater efficacy at higher fixed doses of tirzepatide in non-diabetic adults [5]. SURMOUNT-5 similarly shows that semaglutide MTD outperforms the standard 2.4 mg dose [20], whereas earlier STEP trials suggest semaglutide's efficacy plateaus at 2.4 mg [13,15]. By pooling evidence across randomized trials, this network meta-analysis provides indirect comparisons and treatment rankings that head-to-head studies have yet to deliver [11]. While the superior efficacy of high-dose tirzepatide is clear, it is accompanied by higher rates of gastrointestinal adverse events and treatment discontinuation. Clinicians must therefore balance the magnitude of weight loss against tolerability when choosing among these pharmacologic options.

Mechanistic Insights

The superior weight-loss efficacy of tirzepatide relative to semaglutide likely arises from differences in their pharmacological actions. Semaglutide is a selective GLP-1 receptor agonist that enhances satiety, slows gastric emptying, and reduces energy intake through central and peripheral pathways [22]. Tirzepatide, in contrast, activates both GLP-1 and GIP receptors. GIP receptor engagement has been linked to improved insulin sensitivity, more efficient lipid handling in adipocytes, and additional appetite suppression, acting synergistically with GLP-1 signaling. Pre-clinical and early clinical studies, therefore, suggest that dual agonism can produce larger reductions in energy intake and body weight than GLP-1 agonism alone [23-25]. Broader activation of incretin pathways may also explain the higher rates of gastrointestinal adverse events and treatment discontinuation observed with high-dose tirzepatide. Achieving the MTD for either agent likely reflects near-maximal receptor engagement, which inherently increases the risk of dose-related side effects. *Strengths*

This study has several key strengths. First, it employed a comprehensive network meta-analysis to compare all clinically relevant doses of tirzepatide and semaglutide, including MTD regimens. The inclusion of SUCRA-based rankings allowed for a clear and interpretable hierarchy of efficacy across multiple weight loss thresholds. Additionally, we applied the GRADE framework to evaluate the certainty of evidence for the clinically important outcome of ≥15% weight loss, providing a transparent appraisal of the strength of recommendations based on our NMA results. Second, the consistency of findings across both fixed- and random-effects models reinforces the robustness of the observed dose-response patterns and treatment effects. Third, the assessment of small-study effects and publication bias using comparison-adjusted funnel plots and Egger-type regression revealed no visual or statistical evidence of asymmetry, further supporting the reliability of the results. Finally, the analysis was limited to non-diabetic adults with obesity, the primary population for whom these pharmacologic treatments are indicated, enhancing the clinical relevance and generalizability of the findings to real-world obesity management in this group.

Limitations

These findings should be interpreted in light of several limitations. First, semaglutide at its maximum MTD was examined in only one trial (SURMOUNT-5), limiting the precision and generalizability of its comparative estimates. Second, although the network meta-analysis enabled indirect comparisons, several treatment contrasts lacked direct head-to-head data, introducing additional uncertainty and relying on the assumption of transitivity. Third, despite formal risk of bias assessment, differences in trial duration, participant characteristics, and outcome reporting could still have influenced treatment effects. Fourth, safety outcomes, particularly gastrointestinal adverse events, were reported inconsistently across trials and were therefore not incorporated into SUCRA rankings. Finally, this analysis did not evaluate long-term weight maintenance after treatment cessation, an important consideration for chronic obesity management.

Clinical and Research Implications

This network meta-analysis shows that higher doses of tirzepatide, particularly the 15 mg and MTD regimens, produce the greatest weight loss in non-diabetic adults with obesity. These findings support tirzepatide as a leading pharmacologic option when the therapeutic goal is maximal weight reduction. However, the same higher-dose schedules, along with semaglutide MTD, carry a greater risk of gastrointestinal adverse events and treatment discontinuation. Clinicians must therefore balance efficacy against tolerability and tailor therapy to individual patient preferences, comorbidities, and side-effect profiles through shared decision-making.

Important evidence gaps remain. Few head-to-head trials have compared tirzepatide and semaglutide across their full dose ranges. Future randomized controlled studies should directly contrast equivalent doses, evaluate longer-term outcomes such as weight maintenance durability, and incorporate patient-reported measures, cardiovascular endpoints, and subgroup analyses by sex, age, and baseline BMI. Such data are essential to guide fully informed, evidence-based obesity pharmacotherapy.

Economic Considerations

Both tirzepatide and semaglutide are costly injectable therapies, and real-world access can be constrained by insurance coverage limits, prior-authorization hurdles, and high out-of-pocket costs. These challenges are particularly acute for patients without diabetes, for whom obesity treatment coverage is often inconsistent. As the range of pharmacologic options for obesity grows, cost-effectiveness has become a crucial factor in clinical and policy decisions. A short-term US analysis reported that tirzepatide delivers more weight loss per dollar spent than semaglutide or liraglutide, especially in individuals without diabetes [26]. In contrast, a lifetime cost-effectiveness model found that, at current net prices, neither tirzepatide nor semaglutide meets conventional cost-effectiveness thresholds, despite notable health benefits, underscoring the need for pricing reforms to improve equitable access [27].

These economic considerations may influence treatment selection as strongly as efficacy, particularly for under-insured patients or those reliant on public insurance. Our findings can inform future clinical guidelines and reimbursement policies, suggesting that higher-dose tirzepatide may be prioritized for patients who both need substantial weight reduction and can tolerate its side-effect profile. Ultimately, long-term success in obesity management will hinge on integrating pharmacologic therapy with structured lifestyle interventions, including dietary modification, increased physical activity, and behavioral counseling.

## Conclusions

In this network meta-analysis of non-diabetic adults with obesity, tirzepatide 15 mg and tirzepatide at its MTD produced the largest reductions in body weight and waist circumference, consistently outperforming lower doses of tirzepatide and both doses of semaglutide across categorical weight-loss thresholds (≥5%, ≥10%, and ≥15%). These benefits followed a clear dose-response gradient and remained stable in sensitivity analyses. The greater efficacy of higher doses, however, was offset by higher rates of gastrointestinal adverse events and treatment discontinuation, especially with tirzepatide MTD and semaglutide MTD. These findings highlight the clinical trade-off between maximal weight reduction and tolerability at higher doses of incretin-based therapies. Clinical decision-making should therefore balance these competing considerations and tailor therapy to individual patient goals, comorbidities, and side-effect profiles. The lack of head-to-head trials spanning the full dose ranges of tirzepatide and semaglutide limits direct comparison and highlights an important evidence gap. Economic evaluations further suggest that, at current prices, neither agent is cost-effective over a lifetime horizon, indicating a need for pricing and policy reforms to improve access.

Future research should include long-term trials that directly compare equivalent doses of tirzepatide and semaglutide, evaluate the durability of weight maintenance, capture quality-of-life and cardiometabolic outcomes, and explore subgroup responses. Such evidence will be essential for refining clinical guidelines and optimizing pharmacologic strategies in the treatment of obesity.

## References

[1] (2025). Obesity and overweight. https://www.who.int/news-room/fact-sheets/detail/obesity-and-overweight.

[2] Guh DP, Zhang W, Bansback N, Amarsi Z, Birmingham CL, Anis AH (2009). The incidence of co-morbidities related to obesity and overweight: a systematic review and meta-analysis. BMC Public Health.

[3] Grunvald E, Shah R, Hernaez R (2022). AGA clinical practice guideline on pharmacological interventions for adults with obesity. Gastroenterology.

[4] Busetto L, Dicker D, Frühbeck G, Halford JC, Sbraccia P, Yumuk V, Goossens GH (2024). A new framework for the diagnosis, staging and management of obesity in adults. Nat Med.

[5] Kasagga A, Assefa AK, Amin MN, Hashish R, Agha Tabari K, Swami SS, Nakasagga K (2025). Dose-dependent efficacy and safety of tirzepatide for weight loss in non-diabetic adults with obesity: a systematic review and meta-analysis of randomized controlled trials. Cureus.

[6] Roomy MA, Hussain K, Behbehani HM (2024). Therapeutic advances in obesity management: an overview of the therapeutic interventions. Front Endocrinol (Lausanne).

[7] Page MJ, McKenzie JE, Bossuyt PM (2021). The PRISMA 2020 statement: an updated guideline for reporting systematic reviews. BMJ.

[8] (2024). Cochrane Handbook for Systematic Reviews of Interventions. https://www.cochrane.org/authors/handbooks-and-manuals/handbook.

[9] Sterne JA, Savović J, Page MJ (2019). RoB 2: a revised tool for assessing risk of bias in randomised trials. BMJ.

[10] McGuinness LA, Higgins JP (2021). Risk-of-bias VISualization (robvis): an R package and Shiny web app for visualizing risk-of-bias assessments. Res Synth Methods.

[11] Balduzzi S, Rücker G, Nikolakopoulou A, Papakonstantinou T, Salanti G, Efthimiou O, Schwarzer G (2023). netmeta: an R package for network meta-analysis using frequentist methods. J Stat Softw.

[12] Rücker G, Schwarzer G (2015). Ranking treatments in frequentist network meta-analysis works without resampling methods. BMC Med Res Methodol.

[13] Wilding JP, Batterham RL, Calanna S (2021). Once-weekly semaglutide in adults with overweight or obesity. N Engl J Med.

[14] Wadden TA, Bailey TS, Billings LK (2021). Effect of subcutaneous semaglutide vs placebo as an adjunct to intensive behavioral therapy on body weight in adults with overweight or obesity: the STEP 3 randomized clinical trial. JAMA.

[15] Garvey WT, Batterham RL, Bhatta M (2022). Two-year effects of semaglutide in adults with overweight or obesity: the STEP 5 trial. Nat Med.

[16] Jastreboff AM, Aronne LJ, Ahmad NN (2022). Tirzepatide once weekly for the treatment of obesity. N Engl J Med.

[17] Wadden TA, Chao AM, Machineni S (2023). Tirzepatide after intensive lifestyle intervention in adults with overweight or obesity: the SURMOUNT-3 phase 3 trial. Nat Med.

[18] Zhao L, Cheng Z, Lu Y (2024). Tirzepatide for weight reduction in Chinese adults with obesity: the SURMOUNT-CN randomized clinical trial. JAMA.

[19] Kadowaki T, Kiyosue A, Shingaki T, Oura T, Yokote K (2025). Efficacy and safety of once-weekly tirzepatide in Japanese patients with obesity disease (SURMOUNT-J): a multicentre, randomised, double-blind, placebo-controlled phase 3 trial. Lancet Diabetes Endocrinol.

[20] Aronne LJ, Horn DB, le Roux CW (2025). Tirzepatide as compared with semaglutide for the treatment of obesity. N Engl J Med.

[21] Yepes-Nuñez JJ, Li SA, Guyatt G (2019). Development of the summary of findings table for network meta-analysis. J Clin Epidemiol.

[22] Campbell JE, Drucker DJ (2013). Pharmacology, physiology, and mechanisms of incretin hormone action. Cell Metab.

[23] Finan B, Ma T, Ottaway N (2013). Unimolecular dual incretins maximize metabolic benefits in rodents, monkeys, and humans. Sci Transl Med.

[24] Nauck MA, D'Alessio DA (2022). Tirzepatide, a dual GIP/GLP-1 receptor co-agonist for the treatment of type 2 diabetes with unmatched effectiveness regrading glycaemic control and body weight reduction. Cardiovasc Diabetol.

[25] Coskun T, Sloop KW, Loghin C (2018). LY3298176, a novel dual GIP and GLP-1 receptor agonist for the treatment of type 2 diabetes mellitus: from discovery to clinical proof of concept. Mol Metab.

[26] Liu L, Cui J, Neidecker MV, Nahata MC (2025). Tirzepatide vs semaglutide and liraglutide for weight loss in patients with overweight or obesity without diabetes: a short-term cost-effectiveness analysis in the United States. J Manag Care Spec Pharm.

[27] Hwang JH, Laiteerapong N, Huang ES, Kim DD (2025). Lifetime health effects and cost-effectiveness of tirzepatide and semaglutide in US adults. JAMA Health Forum.

